# Scraps

**Published:** 1894-03

**Authors:** 


### SCRAPS

PICKED UP BY THE ASSISTANT-EDITOR.

---

**Clinical Records** (6).—Doctor to sick man recovering from typhoid: "I think we may venture now to make some further change in your diet. What do you say to having, to-morrow, a little fish?" Patient, with an enormous desire for food: "Oh, doctor, I would very much rather have a *big* fish."

**The Leucocyte's Lament.**—The following has been sent to me by a subscriber who received it direct from Cambridge:

"The leucocyte or white corpuscle is, we have every reason to believe, capable of existence. Some authorities have asserted it is a normal constituent of the blood, but the matter has not been fully worked out, neither is the time ripe for a dogmatic statement."

The leucocyte was in a gland
With inflammation red,
He grasped a comrade by the hand
And with a sob he said:
"'Mid solitary follicles
I wend my weary way,
Deep down in crypts of Lieberkühn
Far far from light of day.
Alas! this aching nucleus
Can ne'er be free from pain,
While tissues hide my beauteous bride
I ne'er shall see again.
A rosy-red corpuscle she,
The pride of all the spleen.
Her like in this dark gland, I fear,
Will never more be seen.
A fierce bacillus captured her,
And reft her from my side;
Carbolic oil his plans did foil,
But ah! it slew my bride.
With pseudopodia feebly bent
And bowed down nucleus, I
Must turn to pus."—And, speaking thus,
He wandered forth to die.
Oh! lightly they'll talk of that leucocyte true
As they label and mount and degrade him,
But little he'll reck, when with aniline blue
They've stained and in Canada laid him.

**Medical Philology** (IX.).—In the *Promptorium* occurs "Bony, or hurtynge," with the Latin equivalent of "Fleumon," taken from Johannes Januensis (1286), and "flegmen" from *Campus florum* (*c.* 1359). In Pynson's printed edition (1499), "tumor" was added to the definitions. Mr. Way's note is:

The *Catholicon* explains *flegmen* to be, "*tumor sanguinis. Item flegmina sunt quando in manibus et pedibus callosi sulci sunt.*" It would appear to be the same as a bunnian, the derivation of which has been traced from the French, "*bigne, bosse, enflure, tumeur.*" ROQUEF. Cotgrave renders it a bump or knob, and he gives also "*Bigne*, club-footed." Sir Thos. Browne, Forby, and Moore, give the word bunny, a small swelling caused by a fall or blow; in Essex, "a boine on the head." In Cullum's Hawsted, among the words of local use, is given bunny, a swelling from a blow.

Cotgrave's full definition of "Bigne" is "A bumpe, knob, rising or swelling after a knocke."

From the first of the equivalents given in the *Promptorium* it would seem that the earliest application of the word was to an inflamed swelling, proceeding, as its alternative of "hurtynge" would show, from an injury. "Bunny" early became one of its alternative spellings. The *New English Dictionary* quotes "Bownche or bunnye, *gibba*," from Huloet's *Abecedarium* (1552), and "Contunial bunnies and looseness of certain joints" (II. cclxxix. 793), from the 1633 edition of Gerard's *Herbal*, first printed in 1597. Prof. Skeat points out that bump, bun, bunch are also all connected with the idea of a rounded lump or knob. Cotgrave gives "Bignets: *Little round loaues, or lumps made of fine meale, oyle, or butter, and reasons: bunnes, Lenten loaues; also, flat fritters made like small pancakes.*" In 1543, Traheron's translation of Vigo's *Works of Chirurgerye* speaks of "the gibbosyte or bounch of the liver" (I. x. 9), and Gerard says, "The leauen made of Wheate . . . openeth all swellings, bunches, tumors and felons" (I. xl. 60).

The present restricted use of the word bunion is of quite recent date. The *New English Dictionary* goes no further than to say that the modern "bunion" is probably connected with "bunny," of which "bony," as it appears in the *Promptorium*, was the earliest form. In case this note should be seen by any non-medical reader, it is perhaps worth while to mention that "bony" has no connection with bone. "Bunnians" was used by Rowe about the beginning of the eighteenth century as a term for corns.

The etymology of these allied words is doubtful. I hope some reader will work out their connection more thoroughly than I have been able to do.

**The Black Death in Chaucer's Time.**—In the *Journal* for September, 1889, a note concerning the Black Death was given from Hecker's *Epidemics of the Middle Ages.* In connection with this, the following passage from Prof. A. W. Ward's *Chaucer*, in the "English Men of Letters" series, will be read with interest: "In the year of King Richard II.'s accession (1377), according to a trustworthy calculation based upon the result of that year's poll-tax, the total number of the inhabitants of England seems to have been two millions and a half. A quarter of a century earlier—in the days of Chaucer's boyhood—their numbers had been perhaps twice as large. For not less than four great pestilences (in 1348-9, 1361-2, 1369, and 1375-6) had swept over the land, and at least one-half of its population, including two-thirds of the inhabitants of the capital, had been carried off by the ravages of the obstinate epidemic—'the foul death of England,' as it was called in a formula of execration in use among the people. In this year 1377, London, where Chaucer was doubtless born as well as bred, where the greater part of his life was spent, and where the memory of his name is one of those associations which seem familiarly to haunt the banks of the historic river from Thames Street to Westminster, apparently numbered not more than 35,000 souls. But if, from the nature of the case, no place was more exposed than London to the inroads of the Black Death, neither was any other so likely elastically to recover from them. For the reign of Edward III. had witnessed a momentous advance in the prosperity of the capital,—an advance reflecting itself in the outward changes introduced during the same period into the architecture of the city. Its wealth had grown larger as its houses had grown higher; and mediæval London, such as we are apt to picture it to ourselves, seems to have derived those leading features which it so long retained, from the days when Chaucer, with downcast but very observant eyes, passed along its streets between Billingsgate and Aldgate. Still, here as elsewhere in England the remembrance of the most awful physical visitations which have ever befallen the country must have long lingered; and, after all has been said, it is wonderful that the traces of them should be so exceedingly scanty in Chaucer's pages. Twice only in his poems does he refer to the Plague:—once in an allegorical fiction which is of Italian if not of French origin, and where, therefore, no special reference to the ravages of the disease *in England* may be intended when Death is said to have 'a thousand slain this pestilence,'—

> he hath slain this year
> Hence over a mile, within a great villáge
> Both men and women, child and hind and page.

The other allusion is a more than half humorous one. It occurs in the description of the *Doctor of Physic*, the grave graduate in purple surcoat and blue white-furred hood; nor, by the way, may this portrait itself be altogether without its use as throwing some light on the helplessness of fourteenth century medical science. For though in all the world there was none like this doctor to *speak* of physic and of surgery;—though he was a very perfect practitioner, and never at a loss for telling the cause of any malady and for supplying the patient with the appropriate drug, sent in by the doctor's old and valued friends the apothecaries;—though he was well versed in all the authorities from Æsculapius to the writer of the *Rosa Anglica* (who cures inflammation homœopathically by the use of red draperies);—though like a truly wise physician he began at home by caring anxiously for his own digestion and for his peace of mind ('his study was but little in the Bible'):—yet the basis of his scientific knowledge was 'astronomy,' i.e., astrology, 'the better part of medicine,' as Roger Bacon calls it; together with that 'natural magic' by which, as Chaucer elsewhere tells us, the famous among the learned have known how to make men whole or sick. And there was one specific which, from a double point of view, Chaucer's Doctor of Physic esteemed very highly, and was loth to part with on frivolous pretexts. He was but easy (i. e. slack) of 'dispence:'—

> He keptë that he won in pestilence.
> For gold in physic is a cordial;
> Therefore he lovèd gold in speciál."

